# A Simultaneous Presentation of Nasopharyngeal Carcinoma and Latent Cervical Tuberculosis: Insights into a Complex Case

**DOI:** 10.3390/diagnostics15030357

**Published:** 2025-02-04

**Authors:** Ingrid-Denisa Barcan, Raluca Maria Closca, Marina Rakitovan, Andreea-Mihaela Banta, Flavia Zara, Sorin Adalbert Dema, Claudia Raluca Balasa Virzob, Ioana Delia Horhat

**Affiliations:** 1 ENT Department, University of Medicine and Pharmacy “Victor Babes”, 300041 Timisoara, Romania; ingrid.barcan@umft.ro (I.-D.B.); andreea.banta@umft.ro (A.-M.B.); delia.horhat@umft.ro (I.D.H.); 2 ENT Department, Emergency City Hospital, 300254 Timisoara, Romania; 3 Department of Microscopic Morphology, University of Medicine and Pharmacy “Victor Babes”, 300041 Timisoara, Romania; marina.rakitovan@umft.ro (M.R.); flavia.zara@umft.ro (F.Z.); 4 Department of Pathology, Emergency City Hospital, 300254 Timisoara, Romania; 5 Angiogenesis Research Center “Victor Babes”, University of Medicine and Pharmacy, 300041 Timisoara, Romania; 6 Oro-Maxillo-Facial Surgery Clinic of the Emergency City Hospital, 300062 Timisoara, Romania; 7 Department of Oncology, University of Medicine and Pharmacy “Victor Babes”, 300041 Timisoara, Romania; dema.sorin@umft.ro; 8 Department of Radiotherapy, Emergency City Hospital, 300595 Timisoara, Romania; 9 Department of Clinic Nursing, University of Medicine and Pharmacy “Victor Babes”, 300041 Timisoara, Romania; virzob.claudia@umft.ro

**Keywords:** nasopharyngeal carcinoma, cervical tuberculosis, lymph node tuberculosis

## Abstract

**Background and Clinical Significance:** Tuberculosis infection triggers a chronic inflammatory response that can create a favorable environment for the development of cancer cells. Chronic inflammation can lead to DNA damage, increased cell proliferation, and impaired immune surveillance. Nasopharyngeal carcinoma is an aggressive malignant tumor with a very poor prognosis, despite the current oncology therapy. **Case Presentation:** The case presents following clinical, imaging, and histopathological aspects, as well as the oncological follow-up of the patient over a period of 8 years. This paper presents the case of a 49-year-old patient diagnosed with cervical lymph node tuberculosis while biopsied for the diagnosis of nasopharyngeal carcinoma with cervical lymph node metastases. **Conclusions:** The overlap of this malignancy with an infection of mycobacterial etiology complicates the outcome of the disease even more, making treatment and prognosis difficult.

## 1. Introduction

Nasopharyngeal cancer (NPC) is a rare malignancy among Caucasians, with an annual incidence of less than 1 case per 100,000 individuals [1]. The pharyngeal recess is the most frequent site of origin for NPC, and the second most common site is the superior posterior wall of the nasopharynx [2]. NPC primarily affects adults (the incidence rises after the age of 30, reaching a peak between 40 and 60 years old), although pediatric cases can also occur [3]. NPC is significantly more common in men than in women, with a male incidence often double or triple those of females [4]. Rosenmüller’s fossa is the site of origin of most NPC because there is the transition of columnar epithelium to the squamous epithelium [5]. The causative carcinogens of NPC are still being studied, but there are various risk factors such as smoking and alcohol consumption, which are likely linked to the development of keratinizing NPC (K-NPC). Also, diets rich in salt and fermented foods, particularly those high in nitrosamines, have been associated with non-keratinizing NPC (NK-NPC) [6]. Certain high-risk HPV types have a potential contribution to a proportion of NPC cases. Similar to HPV-positive oropharyngeal cancers, HPV-related NPC often presents as non-keratinizing [7,8]. Epstein–Barr virus infection has been suggested as an important etiological factor for nasopharyngeal carcinoma [9,10,11]. However, the Epstein–Barr virus itself is not sufficient to cause this type of cancer; other cofactors, such as genetic susceptibility, are involved [10]. NPC carries a 67% survival rate after five years, which can significantly lower depending on factors such as diagnosis at advanced stages and regional lymph node involvement [12]. The presence of distant metastases as well as cervical lymph node involvement confer a significantly poorer prognosis, with a five-year survival rate of 22.3% [8,13,14]. Unlike other head and neck cancers, NPC is mostly treated by radiotherapy, with or without chemotherapy, and with no resection of the primary tumor [4,5].

Mycobacterium tuberculosis infection constitutes a significant global health burden, affecting an estimated quarter of the world’s population [15]. Cervical tuberculous lymphadenitis is a common extrapulmonary manifestation of tuberculosis [16,17]. While screening for latent tuberculosis infection (LTBI) demonstrably reduces the risk of progression to active TB disease, a substantial proportion of LTBI patients remain undiagnosed and, consequently, untreated. TB in the head and neck area typically results from the reactivation of latent TB infection rather than direct oropharyngeal or nasopharyngeal infection [18,19]. TB remains a significant global health challenge. In 2022, an estimated 10.6 million people contracted TB, leading to approximately 1.3 million deaths [17]. Despite ongoing control efforts since the 1990s, countries like China, one of the world’s 30 highest TB burden nations, continue to grapple with the disease [20,21].

The aim of this study was to present the clinical data, the imaging aspects, and the management of a patient with nasopharyngeal carcinoma and metastatic adenopathy, associated with cervical lymph node tuberculosis.

## 2. Case Presentation

### 2.1. Clinical Findings

This article presents the case of a 49-year-old patient diagnosed in Emergency City Hospital Timisoara, Romania, and followed for a period of 8 years in the Otorhinolaryngology (ENT), Radiotherapy and Oncology Departments.

The patient’s first ENT admission was on 17 December 2015. The patient was presented at the ENT Clinic with a right submandibular adenopathy of 30 mm. Clinical examination and nasal endoscopy revealed the hypertrophy of lymphoid tissue in the right nasopharyngeal lateral wall, behind the Rosenmüller’s fossa. The overlying mucosa and the pharyngeal openings of the Eustachian tube had normal aspects. Under local anesthesia with 1% Lidocaine, a biopsy of the nasopharyngeal lymphoid tissue was performed. No intra- or postoperative complications were reported. The harvested fragments were fixed in 10% (*v*/*w*) neutral buffered formalin and sent to the Pathology Service for microscopical examination. The histopathological examination revealed reactive hyperplasia of the lymphoid tissue. The patient was discharged, and no other action was taken.

The second ENT admission was on 13 June 2016. The patient was admitted to the ENT Clinic with bilateral cervical adenopathy, which had been present for 7 months. The clinical examination revealed right cervical adenopathy of 60/50 mm diameter and left cervical adenopathy of 20/20 mm diameter, both mobile with overlying and underlying planes, non-tender spontaneously or on palpation. After preoperative preparation, a biopsy of the right cervical mass was performed under general anesthesia with endotracheal intubation, and the specimens were sent for histopathological examination. The histopathological examination revealed nodular metastasis of a poorly differentiated non-keratinized squamous cell carcinoma, associated with mycobacterial granulomatous inflammation. The patient underwent the Quantiferon test, as well as a bacteriological examination of the sputum. Both tests were negative, and the diagnosis of latent cervical tuberculosis infection was established. The infectious disease physician decided that the patient had a latent cervical tuberculosis infection. Therefore, the patient did not receive antituberculotic treatment.

On 20 July 2016, the patient returned to the ENT Clinic for the third time. The clinical examination and nasal endoscopy revealed a nasopharyngeal mass. Biopsy of the nasopharyngeal mass was performed under local anesthesia with 1% Lidocaine spray with favorable evolution. The harvested specimen was fixed in 10% (*v*/*w*) neutral buffered formalin and sent for histopathological examination. The histopathological examination revealed a poorly differentiated non-keratinized nasopharyngeal squamous cell carcinoma and mycobacterial granulomatous inflammation. The patient was treated for oncological disease, with reduced doses of chemotherapy and radiotherapy, due to his concomitant latent tuberculous infection as presented below.

### 2.2. Histopathological Findings

The harvested specimen was processed with the usual histological technique and stained with hematoxylin and eosin. The histopathological examination was completed with immunohistochemical staining using the following antibodies: anti-cytokeratins AE1/AE3, 5, 7, and 20 and high-molecular-weight cytokeratin, anti-p63, anti-p53 protein, anti-p16 protein, anti-epithelial membrane antigen, anti-leukocyte common antigen, anti-cluster of differentiation 20, anti-CD4, anti-CD8, anti-CD68, anti-CD1a, anti-CD117, and anti-Epstein–Barr virus. The antibodies and the reagents for immunohistochemical staining were purchased from Leica Bio-systems, New Castle, UK, and all steps of the immunohistochemical reactions were performed with the Leica Bond-Max automatic device (Leica Biosystems Melbourne Pty Ltd., Waverley, Australia).

The microscopic examination of the resected piece of nasopharynx revealed large tumor cells with a syncytial growth pattern, intermingled with an increased amount of inflammatory infiltrate predominantly composed of lymphocytes. The tumor cells had scant eosinophilic cytoplasm and round vesicular nuclei with large nucleoli and showed positivity for cytokeratin AE1/AE3, CK 5, p63 protein, and 34βE12 (strong and diffuse reactions), EMA, and p53 protein (moderate and diffuse reactions). Few cells presented a positive nuclear reaction for p53 protein. They were negative for CK7, CK20, p16 protein, and EBV. The inflammatory infiltrate was predominantly composed of B cells positive for CD20, interspersed with CD4- and CD8-positive T cells, and numerous eosinophils, plasma cells, and dendritic cells presenting antigen CD1a and CD68 positive.

**Figure 1:**
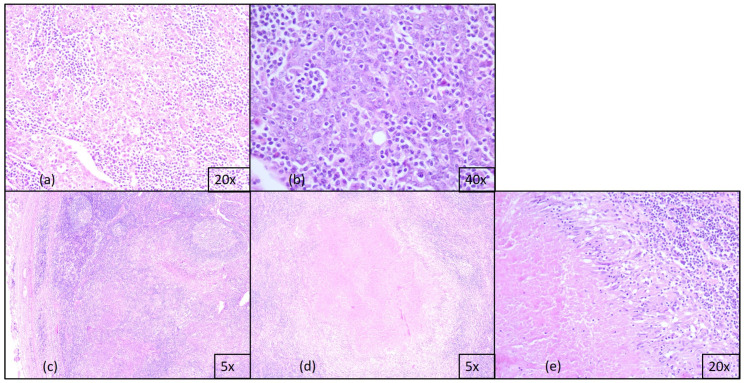
Microscopic aspects of the HE staining slides: (**a**) large tumor cells with a syncytial growth pattern, ob. 20× (**b**) tumor cells intermingled with inflammatory infiltrate, ob. 40×; (**c**) carcinomatous nodular metastasis, ob. 5×; (**d**) cervical lymph node with granuloma, ob. 5×; (**e**) necrotizing epithelioid granuloma with extensive areas of caseous necrosis, ob. 20×.

**Figure 2:**
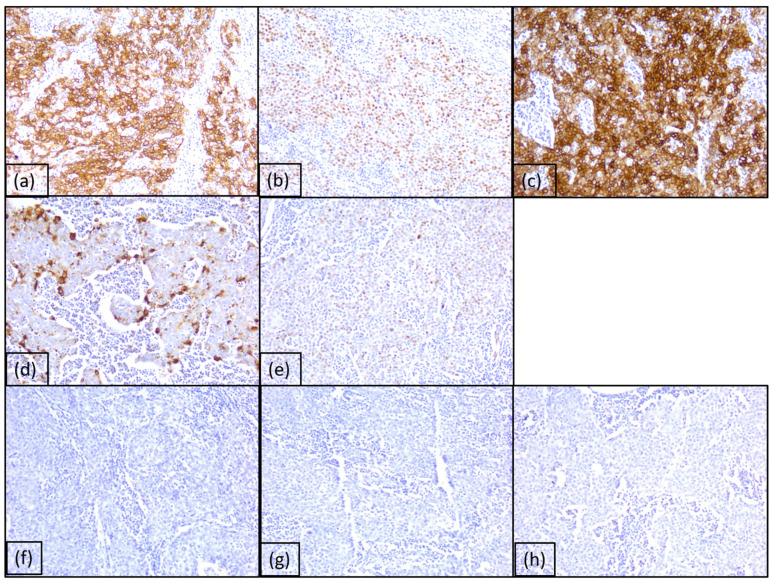
Immunohistochemical profile of tumor cells: (**a**) diffuse and moderate/intense reaction for CK5, ob. 20×; (**b**) diffuse and weak/moderate reaction for p63 protein, ob. 20×; (**c**) diffuse and intense reaction for 34βE12, ob. 20×; (**d**) intense positive reaction for EMA in the marginal areas of the tumor islands, ob. 20×; (**e**) weak positive reaction for p53 protein in 25% of tumor cells, ob. 20×; (**f**–**h**) negative reactions for CK7, CK20, and p16 protein, ob. 20×.

**Figure 3:**
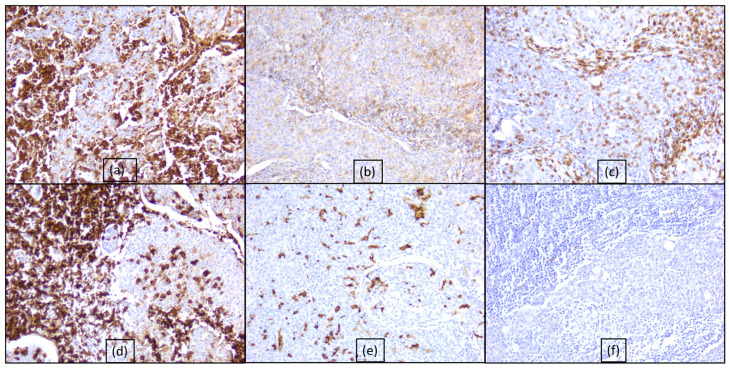
Immunohistochemical profile of inflammatory cells: (**a**) positive reaction of intra- and peritumoral lymphocytes, ob. 20×; (**b**) CD4 positive in T cells, ob. 20×; (**c**) CD8 positive in T cells, ob. 20×; (**d**) CD20 positive in B cells, ob. 20×; (**e**) CD68 positive in dendritic cells, ob. 20×; (**f**) CD117 negative reaction, ob. 20×.

The cervical lymph node presented carcinomatous metastasis and numerous granulomas consisting of epithelioid cells and Langhans cells, with central caseous necrosis. The granuloma showed variable sizes and a tendency to confluence (Figure 1, Figure 2 and Figure 3).

As a result of the histopathological findings, the patient was diagnosed with undifferentiated non-keratinized squamous cell carcinoma of the nasopharynx with metastasis in the cervical lymph nodes. The disease was concomitant with latent tuberculosis of the cervical lymph nodes. Furthermore, the patient was treated for oncological disease, with reduced doses of chemotherapy and radiotherapy, due to his concomitant latent tuberculous infection as presented below.

### 2.3. Oncological Follow-Up

The radiotherapy presentation took place on 8 November 2016. Based on the American Joint Committee on Cancer staging, 8th edition, the cancer stage T3N2Mx, IVA, was established. The patient was admitted to the Radiotherapy Clinic for 66 days and underwent external beam radiotherapy (EBRT) at 6MV energy. The total dose applied to the nasopharynx was 70 GY/35FR/55 days. The total dose applied to the superior, middle, and inferior cervical lymph nodes was 50 GY/25FR/40 days and 6 doses of 35 mg/m^2^ per dose of Cisplatin. The irradiation was well tolerated.

During the patient’s hospitalization in the Radiotherapy Clinic, the following investigations were performed: laboratory tests, ENT and dermatology consultations, and imaging studies (computed tomography (CT) scans, magnetic resonance imaging (MRI), and thoracic X-ray).

#### 2.3.1. Laboratory Findings

Initially, the patient had normal values in his blood tests, but during the chemotherapy, these values decreased. Thus, in the seventh week of chemotherapy, the patient presented leukopenia (WBC = 0.70*10^3^/µL), anemia (RBC = 3.77*10^3^/µL, HCT = 29.5%), and thrombocytopenia (PLT = 175*10^3^/µL). All values normalized by the end of the first cycle of chemotherapy.

#### 2.3.2. ENT and Dermatology Consultations

One month after the initiation of radiotherapy, an ENT consultation was performed. Nasal endoscopy identified a significant reduction in the nasopharynx tumor. Inspection and palpation revealed that the lateral cervical adenopathy decreased in volume.

In the second month of treatment, an ENT consultation confirmed the complete disappearance of the nasopharyngeal mass. Also, the right lateral cervical adenopathy was impalpable at examination.

An ENT consultation confirmed no signs of recurrence ant the 6-month follow-up and one year post-radiotherapy.

A dermatology consultation revealed that the patient had post-medication multiform erythema.

#### 2.3.3. Imaging Findings

**Figure 4:**
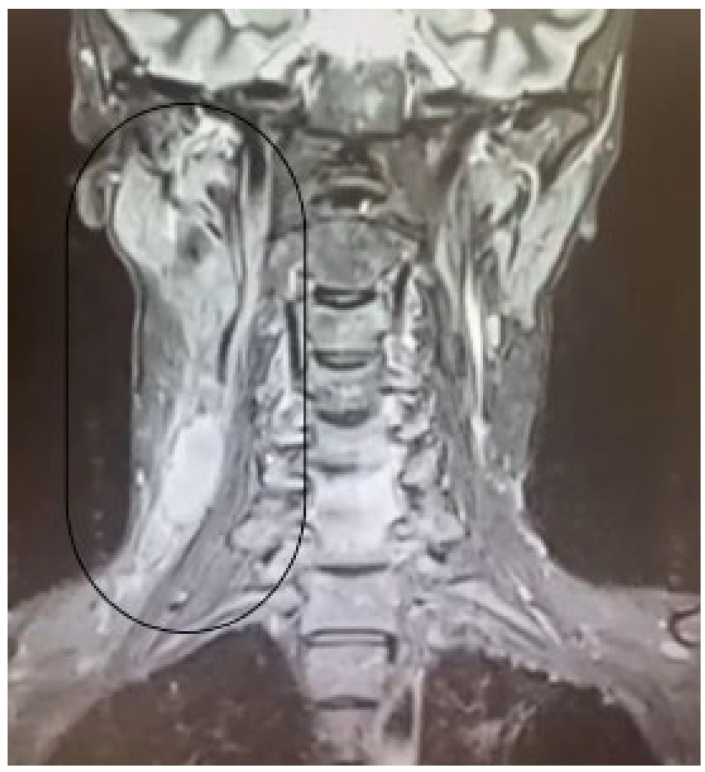
MRI examination of the cervical regions: multiple cervical masses with confluent nodes in the right superior and inferior jugular–carotid groups (circle).

**Figure 5:**
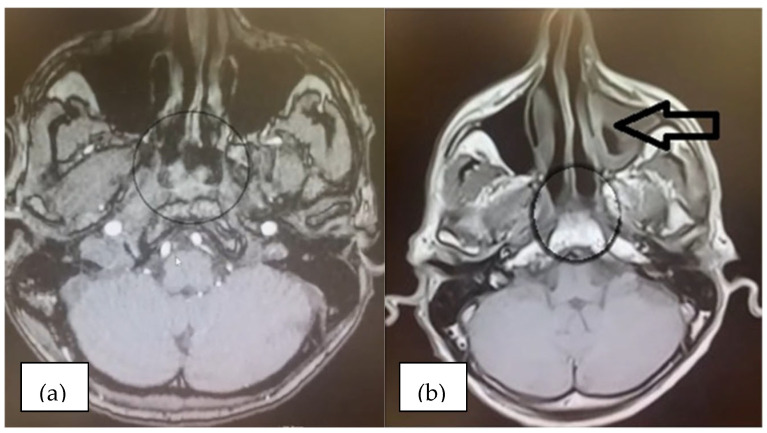
MRI examination of the cerebral regions: aspects before and after chemotherapy: (**a**) initial aspect of the nasopharyngeal tumor mass (circle); (**b**) visible tumor size reduction (circle) with subsequent inflammation of the left maxillary sinus mucosa (arrow).

In 2016, the patient underwent a first contrast-enhanced MRI examination of the cerebral and cervical regions. Multiple bilateral small ischemic lesions were identified, in the frontal supratentorial region, as well as cervical adenopathy with confluent lymph nodes in the following groups: right superior jugular–carotid (13.5/24.5 mm), left superior jugular–carotid (11/18.5 mm), and right inferior jugular–carotid (17/22 mm) (Figure 4). A heterogeneous and asymmetric mass was identified in the nasopharyngeal roof. The tissue mass was 19.5/11 mm in size and crossed the midline, distorting the adenoidal septa (Figure 5a).

In 2017, at the six-month follow-up, contrast-enhanced MRI of the cerebral and cervical region was performed, which showed thickening of the mucosa on the right lateral wall of the nasopharynx in the context of post-radiation changes. The oropharynx, esophagus, and larynx had no pathological changes. No cervical adenopathy was identified. An ENT consultation confirmed no signs of recurrence at 6 months and 1 year post-radiotherapy (Figure 5b).

At the 18-month post-radiotherapy follow-up (2018), contrast-enhanced MRI of the cerebral and cervical region displayed that the cervical larynx and trachea showed no pathological changes at the time of examination but left-sided cervical adenopathy, detected at the posterior cervical level, the largest being approximately 12 mm on the axial plane, with a tendency to confluence. Thoracic X-ray revealed multiple nodular lesions with coastal and subcostal intensity located apically, bilaterally subclavicular, and left mediobasal, more so in the left thorax, with a maximum size of about 15 mm, and these were most likely sequalae lesions.

The 30-month post-radiotherapy follow-up CT scan and contrast-enhanced MRI of the thorax, cerebral, and cervical regions (2019) showed multiple nodular and band-like fibro-calcareous residual lesions in both lungs, predominantly located in the right superior lobe, left superior lobe, and left inferior lobe, associated with pleural attachments, emphysema bullae with a scar-like character and left apical pleuritis, small bronchial dilatations, panlobular changes, and bilateral interstitial septal thickening. In addition, left pleuro-diaphragmatic synechiae were observed. In the nasopharyngeal neoplasm post-radiotherapy, there was slight asymmetry of the nasopharynx and post-radiation inflammatory changes on the left side, without detectable tumor masses at this level. Left cervical adenopathy, with a tendency to confluence, measured up to 20 mm in diameter.

**Figure 6:**
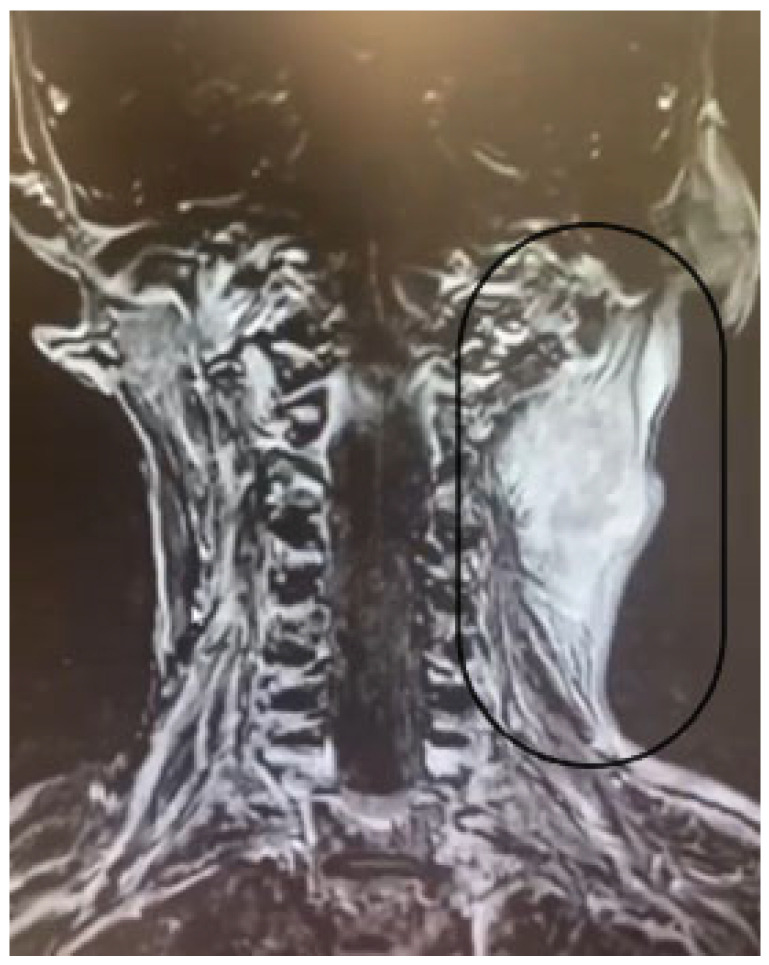
Cervical region MRI: cervical superior confluent lymph node on the left side, tangent to the jugular–carotid vascular bundle and the lower pole of the left parotid gland (circle).

Contrast-enhanced MRI of the cerebral and cervical region (2020) showed a voluminous 45/40 mm cervical superior confluent lymph node on the left side, tangent to the jugular–carotid vascular bundle and the lower pole of the left parotid gland and bilateral apical pulmonary fibrous tracts. A biopsy of the lymph node was performed, and the microscopic examination revealed the presence of nodal metastasis. Due to the metastasis of the left cervical lymph nodes (Figure 6), the patient was admitted to the oncology department for treatment with Doxorubicin 70 mg and Docetaxel 105 mg (AT*6 chemotherapy). The patient tolerated the treatment well.

**Figure 7:**
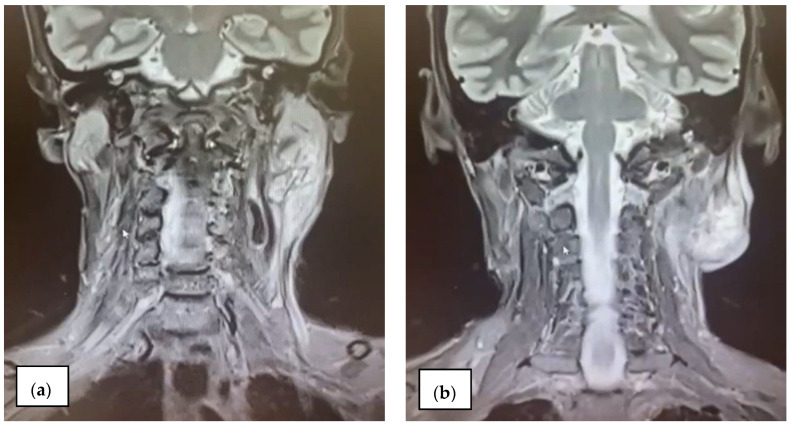
(**a**) Large left cervical lymph node mass; (**b**) enlarged left cervical lymph node block.

In 2021, the patient presented with bulky and fixed left cervical lymphadenopathy. Contrast-enhanced MRI of the cerebral and cervical region revealed a large left cervical mass of 35/28 mm (Figure 7a) and bilateral maxillary sinusitis. Also, a nasopharyngeal mass of 12.7 mm in size, of irregular and heterogeneous appearance, suspicious for recurrence, was identified. A biopsy and histopathological examination were performed, and recurrence was confirmed. It was decided that the patient resumed chemotherapy with Paclitaxel 250 mg and Carboplatin 400 mg (ASC-5). Contrast-enhanced MRI of the cerebral and cervical region at 4 months post-chemotherapy revealed an enlarged left cervical lymph node block of 40/38 mm in diameter and no right cervical lymphadenopathy (Figure 7b).

#### 2.3.4. Outcome

The patient was admitted to the oncology department four months later. Due to the progressive neoplastic disease, the patient received treatment with platinum and fluoropyrimidine. The patient presented cardiac arrest one month after initiation of the treatment.

A flow chart with all admissions as well as the entire oncological follow-up of the case is presented in Table 1.

## 3. Discussion

This case report describes a 49-year-old patient diagnosed with nasopharyngeal carcinoma who developed metastasis in the left cervical lymph nodes and lymph node tuberculosis. The patient underwent radiotherapy and chemotherapy, but the disease still progressed. While the focus was on nasopharyngeal carcinoma, the initial presentation with cervical adenopathy and the later development of pulmonary tuberculous sequelae raise the possibility of lymph node tuberculosis as a potential contributing factor throughout the course of the illness.

Nasopharyngeal carcinoma has a predilection for early and aggressive lymphatic spread [22]. This study exemplifies the typical pattern of regional lymph node metastasis. Early-stage disease often responds well to radiotherapy, as demonstrated in our case [23,24]. However, the subsequent development of recurrent and metastatic disease underscores the aggressive nature of nasopharyngeal carcinoma [25]. The occurrence of metastatic disease highlights the importance of long-term follow-up and surveillance via imaging modalities (MRI and CT scans). The interplay between nasopharyngeal carcinoma and other comorbidities such as tuberculosis further complicates clinical management. The impact of loco-regional metastasis on patients’ prognoses is significant.

The patient’s initial admission with cervical adenopathy and subsequent development of pulmonary tuberculosis sequelae emphasize the importance of considering infectious etiology, such as tuberculosis, in the differential diagnosis of cervical lymphadenopathy [26]. While the definitive diagnosis of nasopharyngeal carcinoma was established, the coexistence of latent nodal tuberculosis significantly complicated the initial diagnostic workup. This case stresses the significance of suspicion for tuberculosis in patients with head and neck masses, especially in endemic regions [27]. According to the European Centre for Disease Prevention and Control, in 2019, Romania was an endemic region for tuberculosis, reporting a male-to-female ratio of 1.8 for new and relapse tuberculosis and high notification rates among children aged between 0 and 14 years [28].

The role of immunosuppression in the development of latent nodal tuberculosis cannot be overstated. Radiotherapy and chemotherapy, while effective in treating nasopharyngeal carcinoma, can significantly compromise immune function, creating a favorable environment for latent tuberculosis reactivation [29]. The temporal relationship between the initiation of the treatment and the emergence of latent nodal tuberculosis supports this possibility. Several mechanisms may explain the association between immunosuppression and tuberculosis reactivation: direct cytotoxic effects, immune dysregulation, and nutritional deficiencies [30]. Direct cytotoxic effects through radiotherapy and chemotherapy induce damage to the immune cells, impairing their ability to control latent tuberculosis [31,32]. Cancer-related inflammation and treatment-induced immune dysfunction can create a good environment for mycobacterial growth, leading to immune dysregulation [33]. Nutritional deficiencies can occur through malnutrition, often associated with advanced cancer and its treatment and can further compromise immune function and increase the risk of infection. Patients with nasopharyngeal carcinoma, especially those undergoing intensive treatment, should be screened for latent tuberculosis infection. Prompt recognition and treatment of tuberculosis reactivation are essential for preventing disease progression and complications. Prophylactic anti-tuberculosis treatment may be considered for high-risk patients [34].

The simultaneous occurrence of nasopharyngeal cancer and lymph node tuberculosis is relatively rare [35,36]. While both conditions can present cervical lymphadenopathy, their coexistence poses a diagnostic challenge. The literature contains a limited number of case reports documenting this unusual association. These cases highlight the importance of considering both malignant and infectious etiologies in patients with cervical lymphadenopathy, especially in regions with a high prevalence of tuberculosis.

The management of patients with metastatic nasopharyngeal carcinoma remains an important clinical challenge. This case report demonstrates the effectiveness of radiotherapy in achieving initial disease control, although the ultimate prognosis is guarded due to the propensity for recurrent and distant metastasis. Collaboration between oncologists, ENT surgeons, radiologists, pathologists, and infectious disease specialists is crucial for optimal patient care. Early and accurate diagnosis and appropriate treatment strategies are essential for improving outcomes.

## 4. Conclusions

This case report illustrates the complex clinical presentation and management of a patient with nasopharyngeal carcinoma, metastatic adenopathy, and lymph node tuberculosis. While advancements in treatment have improved survival rates, the presence of regional lymph node metastasis, as observed in this study, remains a significant prognostic challenge. Incorporating early detection, comprehensive staging, and multidisciplinary treatment approaches is crucial for optimizing patient outcomes.

The diagnostic challenges encountered in this case emphasize the importance of considering infectious etiologies in the differential diagnosis of cervical lymphadenopathy.

This study discloses the potential role of immunosuppression in the reactivation of latent tuberculosis and the need for the vigilant monitoring of patients undergoing cancer treatment.

## Figures and Tables

**Table 1:** The case flow chart with all admissions and the entire oncological follow-up.

17 December 2015
First ENT admission; Nasopharyngeal biopsy; HP: reactive hyperplasia of the lymphoid tissue.
13 June 2016
Second ENT admission; Right laterocervical mass biopsy; HP: nodular metastasis of poorly differentiated non-keratinized squamous cell carcinoma; mycobacterial granulomatous inflammation.
20 July 2016
Third ENT admission; Nasopharyngeal biopsy; HP: poorly differentiated non-keratinized squamous cell carcinoma; mycobacterial granulomatous inflammation.
6 September 2016–11 November 2016
Adjuvant radiotherapy and chemotherapy.
22 September 2016
Cerebral and cervical MRI: cervical adenopathy; confluent laterocervical lymph node.
12 Octomber 2016
ENT consultation: significant reduction in the nasopharynx tumor; decreased cervical adenopathy.
23 February 2018
Contrast-enhanced MRI: left cervical adenopathy; Thoracic X-ray: sequalae bilateral nodular pulmonary lesions.
31 January 2019
Thoracic CT scan: bilateral residual pulmonary lesion, pleural attachments, emphysema bullae, left apical pleuritis, left pleuro-diaphragmatic synechiae; Contrast-enhanced MRI of the cervical region: post-radiation inflammatory changes on the left side, undetectable tumoral masses, left cervical adenopathy.
13 July 2020
Contrast-enhanced MRI of the cerebral and cervical region: left cervical superior confluent lymph node; bilateral apical pulmonary fibrosis; Left cervical superior lymph node biopsy; HP: nodular metastasis of poorly differentiated non-keratinized squamous cell carcinoma.
18 January 2022
Oncology admission; Chemotherapy: Platinum and Fluoropyrimidine.
1 February 2022
The patient presented unresuscitable cardiac arrest.

## Data Availability

The data that support the findings in this study are available from the corresponding author upon reasonable request.
